# Identifying the genetic causes of developmental disorders and intellectual disability in Africa: a systematic literature review

**DOI:** 10.3389/fgene.2023.1137922

**Published:** 2023-05-10

**Authors:** Fiona Baine-Savanhu, Shelley Macaulay, Nadja Louw, Alanna Bollweg, Kaitlyn Flynn, Mhlekazi Molatoli, Patracia Nevondwe, Heather Seymour, Nadia Carstens, Amanda Krause, Zané Lombard

**Affiliations:** ^1^ Division of Human Genetics, National Health Laboratory Service and School of Pathology, Faculty of Health Sciences, University of the Witwatersrand, Johannesburg, South Africa; ^2^ Genomics Platform, South African Medical Research Council, Cape Town, South Africa

**Keywords:** developmental disorder, intellectual disability, genetic disorder, genetic testing, Africa

## Abstract

**Objective:** Genetic variants cause a significant portion of developmental disorders and intellectual disabilities (DD/ID), but clinical and genetic heterogeneity makes identification challenging. Compounding the issue is a lack of ethnic diversity in studies into the genetic aetiology of DD/ID, with a dearth of data from Africa. This systematic review aimed to comprehensively describe the current knowledge from the African continent on this topic.

**Method:** Applicable literature published up until July 2021 was retrieved from PubMed, Scopus and Web of Science databases, following PRISMA guidelines, focusing on original research reports on DD/ID where African patients were the focus of the study. The quality of the dataset was assessed using appraisal tools from the Joanna Briggs Institute, whereafter metadata was extracted for analysis.

**Results:** A total of 3,803 publications were extracted and screened. After duplicate removal, title, abstract and full paper screening, 287 publications were deemed appropriate for inclusion. Of the papers analysed, a large disparity was seen between work emanating from North Africa compared to sub-Saharan Africa, with North Africa dominating the publications. Representation of African scientists on publications was poorly balanced, with most research being led by international researchers. There are very few systematic cohort studies, particularly using newer technologies, such as chromosomal microarray and next-generation sequencing. Most of the reports on new technology data were generated outside Africa.

**Conclusion:** This review highlights how the molecular epidemiology of DD/ID in Africa is hampered by significant knowledge gaps. Efforts are needed to produce systematically obtained high quality data that can be used to inform appropriate strategies to implement genomic medicine for DD/ID on the African continent, and to successfully bridge healthcare inequalities.

## 1 Introduction

Developmental disorders and intellectual disabilities (DD/ID) are among the most common reasons for diagnostic referral in paediatric primary care (Wright et al., 2018). Some cases are due to congenital anomalies being present from birth (also known as birth defects), but others are only recognized once a child misses one or more developmental milestones and presents with developmental delay (Srour and Shevell, 2014). Developmental disorders involve cognitive, emotional and behavioural impairment, and are formally diagnosed in young children who perform two standard deviations below their age-matched peers (American Psychiatric Association, 2013). Intellectual disability is defined as an intelligence quotient of 70 or less, with an onset prior to the age of 18 years (Petersen et al., 1998). DD/ID persist throughout an individual’s lifetime and require on-going intervention, placing significant pressure on the healthcare system.

Although these disorders are usually individually rare, the cumulative burden is significant; it is estimated that 6% of the global population is affected (Maulik et al., 2011). The burden of DD in low-and middle-income countries (LMIC) is systematically increasing, due to effective prevention and treatment of infectious diseases leading to a significant decrease in childhood mortality over the last two decades (Newton, 2012). There is however a clear gap in knowledge around the epidemiology and aetiology of DD/ID in Africa (Bakare and Munir, 2011; de Vries et al., 2013).

Although the underlying causes vary, up to 80% of DD/ID cases are suspected to have a genetic origin (Amberger et al., 2015). In South Africa, for example, 1 in 15 births is estimated to be affected by a congenital anomaly, of which 80.5% are expected to be genetic, or partially genetic, in nature (Malherbe et al., 2015). Genetic conditions have a clear impact on child mortality and morbidity as well as on medical resources. Many do not have a cure and may require ongoing management; underscoring the need to prioritize research and diagnostic services for these conditions on the African continent. A full understanding of the genetic causes that explain the observed clinical features impacts on the accuracy of diagnosis and optimizes safe medical practice (Wright et al., 2018), avoiding unnecessary investigation and providing accurate information for future family planning.

DD/ID syndromes are particularly difficult to diagnose, due to a high level of phenotypic and genetic heterogeneity. This is compounded in Africa, due to the lack of both dysmorphology and genomic data in public repositories (Lumaka et al., 2022). Elucidating the genetic aetiology of DD/ID is an active area of research and many more disease-causing genes remain to be discovered. New technologies, such as next-generation sequencing, have resulted in improved genetic testing and diagnostic yield, with the American College of Medical Genetics and Genomics (ACMG) now strongly recommending exome sequencing as a first- or second-tier test for patients with DD/ID (Manickam et al., 2021). Noteworthy, is the effort led by the Deciphering Developmental Disorders (DDD) study, that has demonstrated the value of adopting a comprehensive, genome-wide strategy, to clarify the underlying causes of DD/ID (Wright et al., 2015). The DDD study has become a model for the rapid adoption of new technologies in genomic medicine, and the importance of harmonized phenotyping and data sharing in disease-gene discovery. The study recruited >33,500 individuals from families with severe, likely monogenic developmental disorders from 24 regional genetics services around the United Kingdom and Ireland, and led to the development of sophisticated phenotyping tools, clinical decision support software, an extensive knowledgebase on DD/ID and practical bioinformatics processes for data analysis and interpretation (Firth et al., 2009; Deciphering Developmental Disorders, 2015).

Despite such global advances, the lack of data from African populations remains apparent–as few as 22% of participants in genomics research studies are of non-European ancestry, and most available genetic data come from just three countries - the United Kingdom (40%), United States (19%) and Iceland (12%) (Mills and Rahal, 2019; Tucci and Akey, 2019). Although efforts exist to address this gap, such as the Human Heredity and Health (H3Africa) Consortium (Rotimi et al., 2014), progress has been particularly slow in the rare diseases field, which includes DD/ID disorders (Lumaka et al., 2022). Genetic conditions have not been systematically researched or documented in African patients (Krause et al., 2018), apart from a few common disorders, such as sickle cell anaemia (Tshilolo et al., 2009; Makoni, 2021) and oculocutaneous albinism type 2 (Kromberg and Kerr, 2022), Further, in addition to genomic research, clinical genetics services and expansive healthcare systems are needed to reduce the significant genomic health equity gap (Kamp et al., 2021).

In this systematic review, we aimed to comprehensively evaluate and summarise the existing knowledgebase on genetic conditions that present with DD/ID as major features in African patients. We interrogated the clinical presentation of the African DD/ID patient and the type of diagnostic tools implemented, in order to uncover what the genomic medicine landscape looks like on the African continent.

## 2 Methods

### 2.1 Search strategy and literature selection

We performed a systematic review following the Preferred Reporting Items for Systematic Reviews and Meta-Analyses (PRISMA) guidelines (Moher et al., 2009). We used Search Builder version 1.0 (Kamdar et al., 2015) to assemble search queries for PubMed, Scopus and Web of Science databases. The search terms “genetic”, “chromosome”, “birth defect”, “congenital”; were combined with “dysmorphology”, “intellectual disability”, “developmental delay” and “mental retardation”. Regarding the latter terminology, it should be noted that in recent time, there has been a conscious shift in terminology used to describe ID, which was previously commonly referred to as “mental retardation”. This term has been deemed scientifically inappropriate and socially harmful, but does still persist in some medical publications (Nash et al., 2012). To limit the search to papers describing patients from continental Africa, the names of all 54 African countries were included in the search string. The full search string is provided in Supplementary Material. Searches were performed across the three selected databases in August 2021. Databases were exhaustively searched, and no earliest date limitations were set, resulting in a dataset representing publications from May 1964—July 2021. Where possible, filters were set for human studies and limited to English, Afrikaans and French publications. The online application Rayyan (Ouzzani et al., 2016) was used to remove duplicates. Two authors used Rayyan to independently screen the titles and abstracts of the articles retrieved and resolved disagreements by consensus.

The inclusion criteria to select publications for this systematic review were as follows: original research articles published in a peer-reviewed journal; study participants defined as originating from an African country; phenotype presenting with ID or DD (or mental retardation); a clinical diagnosis of a known genetic syndrome and/or molecular confirmation of a genetic cause underlying the DD/ID phenotype. We did not exclude studies performed outside of Africa as we anticipated a significant proportion of the research to have been performed in collaboration with international institutions (Munung et al., 2017). We excluded articles with a potential environmental influence for the DD/ID phenotype (including alcohol, infection and drug use). For articles where relevance was not clear from the title and abstract screen, the full text publication was downloaded and individually reviewed. This project was not prospectively registered with PROSPERO; a protocol was developed during the planning process.

### 2.2 Data extraction

A list of the articles included in this systematic review is provided in Supplementary Table S1, with the type of information extracted from each article described in Supplementary Table S2. For some analyses, data from North Africa were analysed separately. Many North African countries are defined as upper middle income or high income countries by the World bank (https://www.worldbank.org/), and the region generates one-third of Africa’s total gross domestic product (Dieteren and Bonfrer, 2021). Therefore we wanted to investigate if trends are different to the rest of Africa. We used the African Union regional definitions (African Union, 2022) to designate North African countries as Algeria, Egypt, Libya, Mauritania, Morocco, Sahrawi Arab Democratic Republic, and Tunisia.

There is some redundancy in reported numbers, due to a subset of publications describing more than one type of disorder and/or method of testing. Publications were categorised based on the Strengthening the Reporting of Observational Studies in Epidemiology (STROBE) guidelines (von Elm et al., 2008) as case reports (reports on an individual patient with DD/ID), case series (reports on a group of DD/ID patients with common characteristics), cohort studies (temporal observational studies reporting on DD/ID), prevalence studies (i.e., cross-sectional studies designed to determine prevalence) and case-control studies.

### 2.3 Quality assessment

The quality of each study was assessed using critical appraisal tools from the Joanna Briggs Institute (JBI) (Jordan et al., 2019). Several different types of studies were identified in the search, necessitating the use of type-specific checklists, i.e., for case-control studies, prevalence studies, case reports and case series. The checklists were accessed online at: https://jbi.global/critical-appraisal-tools. Each study was assessed independently by two authors using the relevant checklist and assigned an overall quality score: “good”, “average” or “poor”. Discrepancies were resolved through discussion and articles that were deemed average or poor using the appraisal tools were excluded.

## 3 Results

### 3.1 Literature selection and quality assessment

**FIGURE 1:**
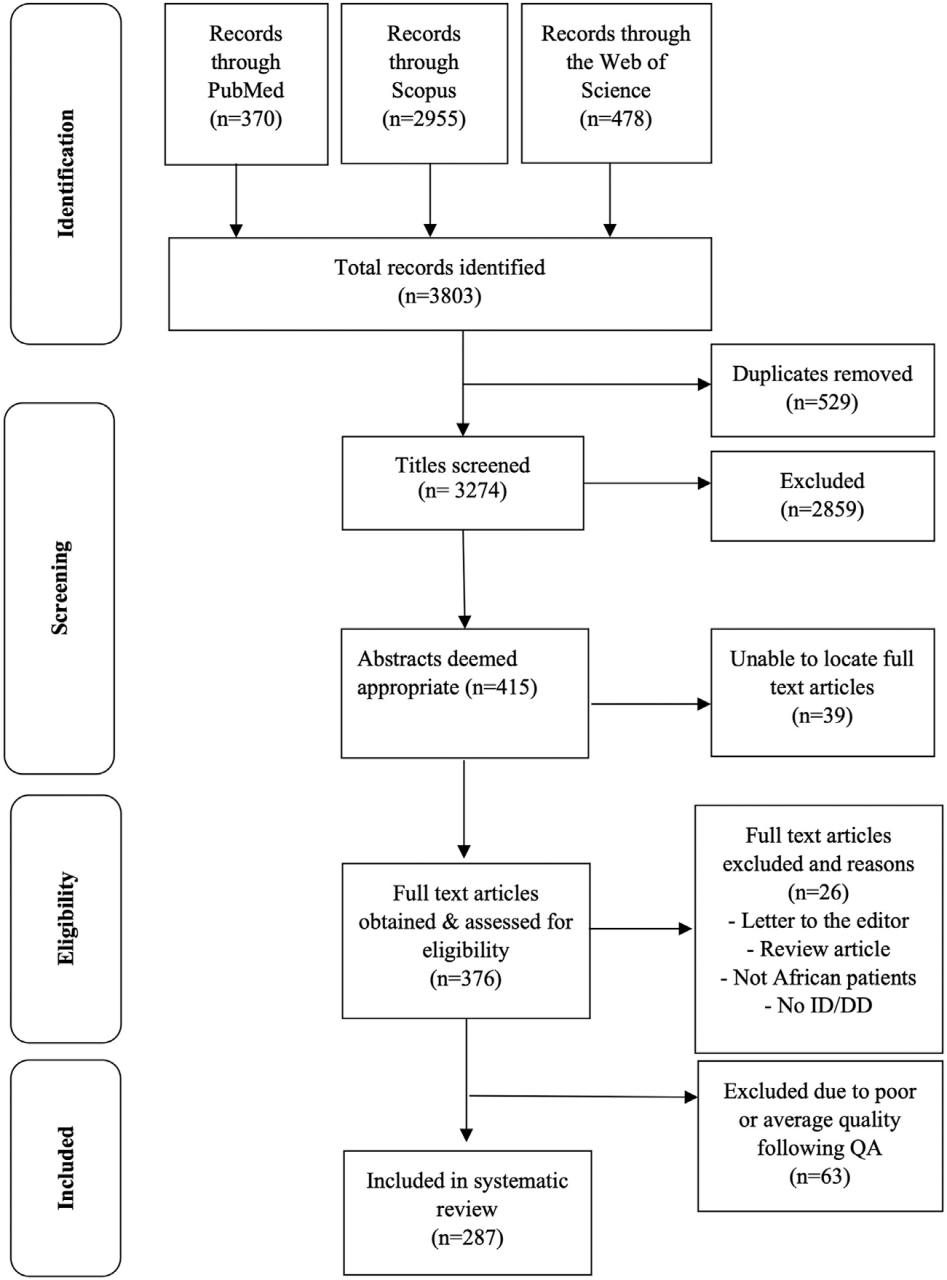
PRISMA flow chart of article selection for the systematic review.

The three databases searched identified 3,803 records of which 529 were duplicates that were removed, resulting in 3,274 unique records. By screening the titles of the publications extracted 2,859 records were excluded based on irrelevance to the research question. The abstracts of 415 records were reviewed and 376 full-text articles obtained and read; including 17 French articles and one Afrikaans article, which were translated using Google Translate (https://translate.google.com/ accessed 31 August 2021). Twenty-six articles were excluded, based on these papers not describing original research (i.e., review articles and letters to the editor), those describing patients who are not continental Africans (e.g., African Americans), or those not including patients with ID/DD. A further 63 articles were excluded following quality assessment, leaving a final total of 287 articles for the systematic review (Figure 1). Of these 287 publications, 160 (55.8%) were case reports, 101 (35.2%) were case series reports, 21 (7.3%) were cohort studies, four (1.4%) were prevalence studies and only one (0.3%) reported a case-control study.

### 3.2 Characteristics of patient cohorts

In line with the observation that DD/ID are generally diagnosed in childhood, most of the studies focused on paediatric cases (92.3%; 265/287). Of these, 40 also reported on adult patients; whereas only a small fraction of publications focused solely on adult patients (5.2%; 15/287) and prenatal cases (1.7%; 5/287).

The dataset included a wide range of conditions that were grouped into four categories, i.e., chromosomal abnormalities (including copy number variants, large structural rearrangements, and aneuploidies); single gene disorders (including small variants, repeat expansions, and clinically recognisable disorders caused by variants in a single gene); conditions of “other” genetic aetiology such as imprinting disorders and mitochondrial diseases; and lastly conditions with unknown genetic aetiology. Most reports focused on targeted investigations of single gene disorders (60.5%) and chromosomal abnormalities (28.9%), which aligned with the testing strategies implemented. Of the five publications on prenatal subjects, one was a case report of unusual findings in a fetus with trisomy 18 (South Africa); one reported the occurrence of trisomy 21 in autopsied fetuses (Tunisia); and three Egyptian publications correlated the molecular and prenatal ultrasound findings in fetuses diagnosed with genetic conditions.

It is well-known that the offspring of consanguineous unions may be at increased risk of genetic disorders (Oniya et al., 2019). Consanguinity was commonly reported (46.3%; 133/287); with the largest proportion of these studies from North Africa, specifically Egypt (103/133; 77.4%), Tunisia (52/133; 39.0%) and Morocco (31/133; 23.3%).

This collection of research provided some novel and interesting observations. A number of publications (18.5%, *n* = 53) reported the discovery of new causative variants in known disease genes, or expanded the phenotype of a known disorder by describing African patients. Examples include novel mutations underlying Noonan syndrome in African patients (Tekendo-Ngongang et al., 2019); and unique presentation of anomalies associated with Muenke syndrome (Abdel-Salam et al., 2011). Most case reports and case series reported a DD/ID phenotype for the first time in an African patient, with 14 publications (mostly from North Africa) describing founder mutations for DD/ID syndromes.

### 3.3 Contributions from Africa

**FIGURE 2:**
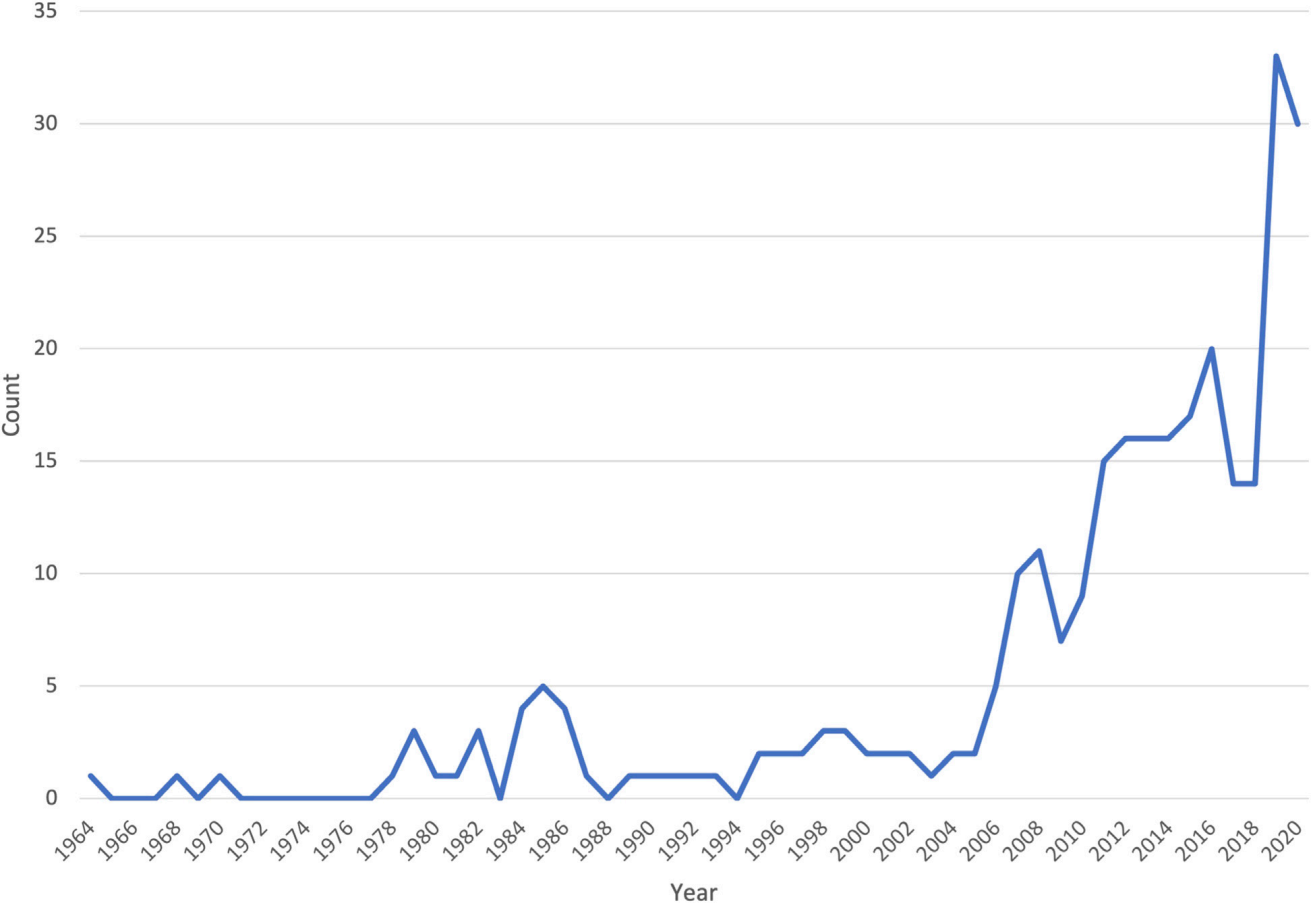
A line graph showing the number of original research articles included in the systematic review, by year of publication.

**FIGURE 3:**
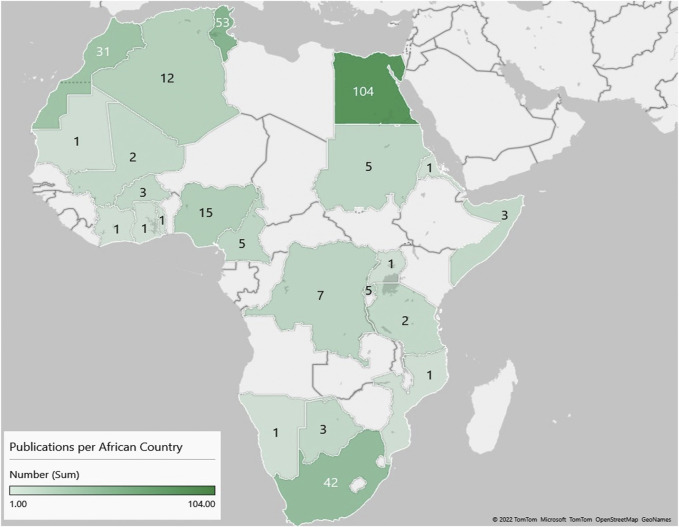
A heat map showing the number of original research publications from Africa, by country, on the genetics of developmental disorders and intellectual disability.

Similar to global trends in medical genomics, there was a marked increase in the number of publications from Africa in the last decade (Figure 2). However, of the 54 countries in Africa, only 23 were represented in the publications reviewed (Figure 3); with more than half of these countries (56.5%, 13/23) having only three or fewer publications. Some publications presented data from more than one country (country count = 296). North Africa dominated the publications from the continent–Egypt had by far the largest output (35.1%, *n* = 104), followed by Tunisia (17.6%, *n* = 52) and Morocco (10.1%, *n* = 30). Outside the North African region, South Africa had the most publications (14.2%, *n* = 42), but a significant proportion of these (*n* = 28/42) focused on patients of European descent. Together, these top four countries represent 77% of the publications included in this review.

When analysing the level of involvement of scientists working in African countries in this set of studies, we again observed different trends in North Africa to the rest of Africa. Whilst publications from North Africa were mostly led by researchers with an affiliation from that region (both as first and last author), in the rest of Africa, 30.9% (29/94) of studies did not include a senior author with affiliation to an African institution, and 14.9% (14/94) of papers also did not feature a first author African researcher. This subset also includes five publications where no authors with an African institutional affiliation were represented on the paper, despite the study cohort being from Africa.

### 3.4 Genetic testing capacity in Africa

Most publications (72.8%, 210/287) included efforts to molecularly confirm the clinical diagnosis made. Of the 77 studies that did not report a molecular genetic finding, 74% (57/77) focused on a well-known single gene disorder generally diagnosed clinically (e.g., Neurofibromatosis), or based on a biochemical test result (e.g., Phenylketonuria).

**FIGURE 4:**
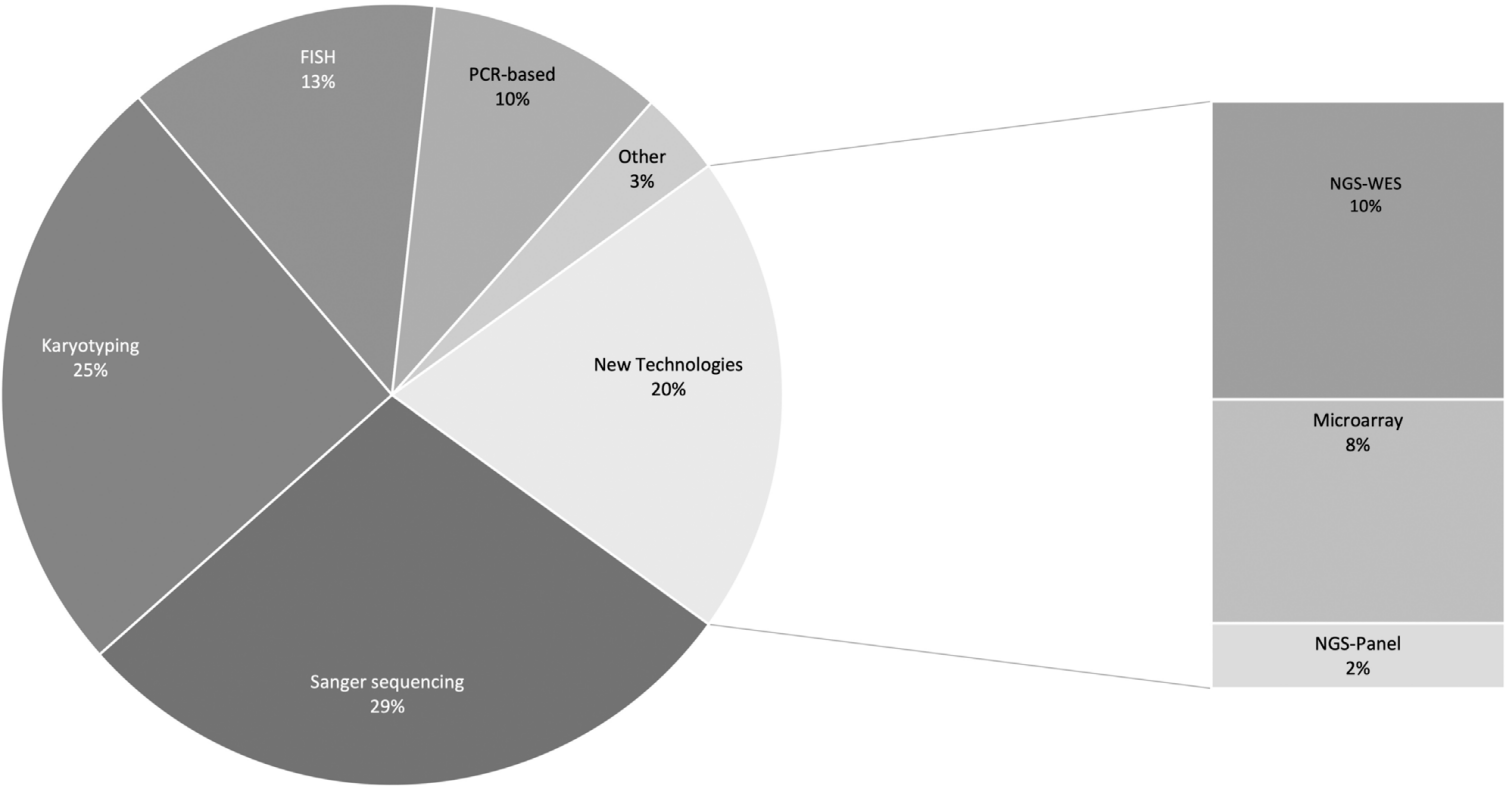
Different molecular testing approaches used to confirm clinical diagnoses. PCR-based analyses include: multiplex ligation-dependent probe amplification, restriction fragment length polymorphism analysis, amplification-refractory mutation system, and real-time PCR, capillary electrophoresis, PCR-denaturing gradient gel electrophoresis, denaturing high-performance liquid chromatography, quantitative multiplex PCR of short fluorescent fragments, high resolution melt analysis, reverse transcription PCR. Other includes: methylation analysis, Southern blot, biochemical test, and mass spectrometry. FISH, fluorescence in situ hybridisation; PCR, polymerase chain reaction; NGS, next-generation sequencing; WES, whole exome sequencing.

An overview of the different laboratory testing approaches employed is shown in Figure 4. Some publications reported more than one type of test (*n* = 316 tests reported in total). Sanger sequencing (90/316, 28.5%) and karyotyping (80/316, 25.3%) were most often used as confirmatory testing approaches. Very few studies (61/287; 21.2%) reported using newer technologies such as next-generation sequencing (NGS) and chromosomal microarray (CMA). In most of this subset of publications (35/61; 57.3%) NGS testing was performed elsewhere in the world and not at an African institution. Dissecting this figure further, we see that studies that reported using new technologies locally were almost exclusively from North Africa–only two of these studies were from sub-Saharan Africa–where NGS was performed in South Africa.

## 4 Discussion

The results from this systematic review have highlighted the gap in knowledge discovery from the African continent regarding the genetic aetiology of DD/ID. The review shows that there are pockets of clinical expertise across the continent with good representation of case identification and reporting, but a lack of capacity for discovery and genetic service delivery still largely exists. The sub-Saharan region of Africa is strikingly underrepresented in this area of research.

The lack of diversity in genomic studies, and the paucity of African genomic data in particular, is now well documented (Bentley et al., 2017; Sirugo et al., 2019). This not only exacerbates health disparities but hampers scientific discovery (Choudhury et al., 2020; Mahajan et al., 2022). Our systematic review demonstrates that this unequal knowledge contribution extends to the DD/ID literature. The vast majority of existing knowledge on the genetic underpinnings of DD/ID in African populations comes from only six countries, i.e., Egypt, Tunisia, South Africa, Morocco, Nigeria, and Algeria. There is no representative scientific literature from 63% of African countries. In countries like South Africa, patients included may not represent all ethnicities equally and may be biased to groups that were historically advantaged in healthcare access. As a consequence the global health disparity gap could significantly widen, as precision medicine moves to the forefront of healthcare (Doble et al., 2017).

A limited number of the studies from this systematic review were broad-based and unbiased in the conditions studied–most target previously reported genetic variants, missing an opportunity for new variant discovery. This implies that the current knowledgebase does not accurately capture the expanded genetic diversity of the African DD/ID patient, a significant oversight given that African genomes represent the greatest genomic diversity globally (Sherman et al., 2019). Increasing genetic studies from Africa will not only hold benefit for African patients, but also for the global community. This was demonstrated persuasively by the H3Africa consortium (Choudhury et al., 2020), who showed that adding whole genome data from a mere 426 African individuals allowed the reclassification of likely pathogenic variants recorded in the ClinVar database due to their highly observed frequency in that African dataset.

Recognising a dysmorphology pattern and accurately linking it to a genetic syndrome is a major diagnostic challenge in DD/ID. The paucity of specialist health services, and particularly clinical genetic services, means that many patients with ID/DD will not even be recognised as requiring investigation (Kamp et al., 2021; Lumaka et al., 2022). Clinical genetics has evolved to incorporate facial dysmorphology analysis technology (Mensah et al., 2022) and “genotype-first” diagnostic strategies (Manickam et al., 2021) through the implementation of powerful genomic sequencing technologies to address this challenge. Facial dysmorphology analysis also performs less well in African populations as patients are poorly represented in training databases (Lumaka et al., 2017), further highlighting the importance of expanding the phenotypes of DD/ID disorders with African data.

The slow adoption of new technologies such as NGS and CMA on the African continent, especially in sub-Saharan Africa, is a cause for concern. CMA has been the internationally recommended first-tier clinical diagnostic test for individuals with DD/ID for over a decade (Miller et al., 2010), but this systematic review has shown that this testing strategy is not yet routinely offered to African patients. The use of limited testing strategies implies that many diagnoses are not being made for African DD/ID patients, rendering the global knowledge of DD/ID aetiology incomplete, and resulting in many African patients remaining undiagnosed. Conditions described are those detectable by older techniques, for example, karyotyping or Fragile X syndrome testing. Patients who tested negative through these traditional limited techniques are generally not followed up with a test with greater resolution or that surveys the genome more comprehensively, implying a bias in reporting. Further to this, through this limited approach, *de novo* mutations are almost certainly missed, which further limits the comprehensive description of the DD/ID mutational spectrum in the African patient, given the prominent role this class of variants play in DD/ID aetiology (Acuna-Hidalgo et al., 2016; McRae et al., 2017).

Globally, the characterization of the molecular causes of DD/ID remains incomplete. Adding data from globally diverse cohorts, especially from Africa will enable discoveries that have global impact (e.g., by refining variant interpretation) as well as local impact (e.g., identifying unique or high frequency variants in African cohorts). Such studies will also be required to develop cost-effective and targeted local approaches. The Deciphering Developmental Disorders in Africa study (DDD-Africa; https://h3africa.org/index.php/ddd-africa/) is one such initiative that aims to improve the scope and delivery of diagnostic and clinical genetic services in Africa, by systematically describing and defining the causes of DD/ID in Africa. This collaborative project has led to the establishment of a rich resource with detailed clinical and phenotype data, whole exome sequencing data and banked DNA samples, from 500 patients and their parents. This is one of very few efforts on the continent, and currently only includes patients from South Africa and the Democratic Republic of Congo, underscoring the need to accelerate broader access to genomics for global health. DDD-Africa has yielded early diagnostic advances for patients (Flynn et al., 2021) and certainly lays the groundwork for future collaborations to enable the promotion and implementation of genomic medicine more broadly across the continent.

Very few prenatal studies were identified through this systematic review (1.7%; 5/287). Diagnosis of chromosomal abnormalities in fetuses remains an important approach to improving the prognostic information that can be provided to parents. Prior to the adoption of any new diagnostic testing strategy, studies must demonstrate clinical and analytical validity (Lord et al., 2019), as well as economic viability (Kodabuckus et al., 2020) in the local context. Improving research outputs in this area could inform the prevention of congenital disorders and positively influence the urgent need to develop and implement policy actions on prenatal testing in sub-Saharan Africa (Darmstadt et al., 2016).

The knowledge gaps uncovered through this systematic review reflect an underinvestment in genomics research -infrastructure and expertise on the continent. A recent report from the World Health Organization (WHO) underscores the importance of genomics to future global health (WHO Science Council, 2022). The report recognizes the global gaps in genomics research and healthcare capabilities, and suggests strategies to overcome these, emphasizing partnership models involving governments, academia, industry, and civil society. Our systematic review emphasises that there is a promising core of expertise around the clinical recognition of DD/ID on the African continent; this is critical to driving genomic medicine efforts locally (Kamp et al., 2021).

This systematic review highlights the paucity of African data on the genetics of DD/ID. Literature on the subject has matured to large evaluations of exome- and genome-based approaches linked to sophisticated computational approaches to improve diagnostic yield and the clinical management of DD/ID in high-income countries (Turro et al., 2020; Gardner et al., 2021; van der Sanden et al., 2022). This review shows that the bulk of African literature is still focussed on small cohorts with single-gene resolution using older, less comprehensive technologies. Reports are largely anecdotal and opportunistic. Efforts are needed to produce systematically obtained high quality data that can be used to inform appropriate strategies to implement genomic medicine for DD/ID on the African continent. This is a crucial focus area to ensure that the promise of health improvement through global health genomics is realised.

## Data Availability

The data analyzed in this study is subject to the following licenses/restrictions: The extracted dataset is available on request. The raw data supporting the conclusion of this article will be made available by the authors, without undue reservation. Requests to access these datasets should be directed to ZL, zane.lombard@wits.ac.za.
