# Supplementary material for: Identifying the genetic causes of developmental disorders and intellectual disability in Africa: a systematic literature review
Source: Front Genet. 2023 May 10;14:1137922. doi: 10.3389/fgene.2023.1137922 (PMC10208355; doi:10.3389/fgene.2023.1137922)
Supplement: Supplementary file 3 [file DataSheet1.docx]

The following search strings were used to search in different databases to compile the comprehensive list of literature reviewed in this paper.

**Scopus and Web of Science**

('Africa' OR 'Algeria' OR 'Angola' OR 'Benin' OR 'Botswana' OR 'Burkina Faso' OR 'Burundi' OR 'Cabo Verde' OR 'Cameroon' OR 'Central African Republic' OR 'Chad' OR 'Comoros' OR 'Congo' OR 'Côte d'Ivoire' OR 'Democratic Republic of the Congo' OR 'Djibouti' OR 'Egypt' OR 'Equatorial Guinea' OR 'Eritrea' OR 'Ethiopia' OR 'Gabon' OR 'Gambia' OR 'Ghana' OR 'Guinea' OR 'Guinea-Bissau' OR 'Kenya' OR 'Lesotho' OR 'Liberia' OR 'Libya' OR 'Madagascar' OR 'Malawi' OR 'Mali' OR 'Mauritania' OR 'Mauritius' OR 'Morocco' OR 'Mozambique' OR 'Namibia' OR 'Niger' OR 'Nigeria' OR 'Rwanda' OR 'São Tomé and Príncipe' OR 'Senegal' OR 'Seychelles' OR 'Sierra Leone' OR 'Somalia' OR 'South Africa' OR 'South Sudan' OR 'Sudan' OR 'Swaziland' OR 'Togo' OR 'Tunisia' OR 'Uganda' OR 'Tanzania' OR 'Zambia' OR 'Zimbabwe' OR 'Cape Verde' OR 'République centrafricaine ' OR 'Ivory Coast' OR 'Zaire' OR 'DRC') AND ('genetic' OR 'chromosome' OR 'birth defect' OR 'congenital') AND ('intellectual disability' OR 'developmental delay' OR 'mental retardation' OR 'dysmorphology')

**PubMed**

("Africa"[All Fields] OR "Algeria"[All Fields] OR "Angola"[All Fields] OR "Benin"[All Fields] OR "Botswana"[All Fields] OR "Burkina Faso"[All Fields] OR "Burundi"[All Fields] OR "Cabo Verde"[All Fields] OR "Cameroon"[All Fields] OR "Central African Republic"[All Fields] OR Chad[All Fields] OR "Comoros"[All Fields] OR "Congo"[All Fields] OR "Côte d'Ivoire"[All Fields] OR "Democratic Republic of the Congo"[All Fields] OR "Djibouti"[All Fields] OR "Egypt"[All Fields] OR "Equatorial Guinea"[All Fields] OR "Eritrea"[All Fields] OR "Ethiopia"[All Fields] OR "Gabon"[All Fields] OR "Gambia"[All Fields] OR "Ghana"[All Fields] OR "Guinea"[All Fields] OR "Guinea-Bissau"[All Fields] OR "Kenya"[All Fields] OR "Lesotho"[All Fields] OR "Liberia"[All Fields] OR "Libya"[All Fields] OR "Madagascar"[All Fields] OR "Malawi"[All Fields] OR "Mali"[All Fields] OR "Mauritania"[All Fields] OR "Mauritius"[All Fields] OR "Morocco"[All Fields] OR "Mozambique"[All Fields] OR "Namibia"[All Fields] OR "Niger"[All Fields] OR "Nigeria"[All Fields] OR "Rwanda"[All Fields] OR "São Tomé and Príncipe"[All Fields] OR "Senegal"[All Fields] OR "Seychelles"[All Fields] OR "Sierra Leone"[All Fields] OR "Somalia"[All Fields] OR "South Africa"[All Fields] OR "South Sudan"[All Fields] OR "Sudan"[All Fields] OR "Swaziland"[All Fields] OR "Togo"[All Fields] OR "Tunisia"[All Fields] OR "Uganda"[All Fields] OR "Tanzania"[All Fields] OR "Zambia"[All Fields] OR "Zimbabwe"[All Fields] OR "Cape Verde"[All Fields] OR "République centrafricaine "[All Fields] OR "Ivory Coast"[All Fields] OR "Zaire"[All Fields] OR "DRC"[All Fields]) AND ("genetic"[All Fields] OR "chromosome"[All Fields] OR "birth defect"[All Fields] OR "congenital"[All Fields]) AND ("intellectual disability"[All Fields] OR "developmental delay"[All Fields] OR "mental retardation"[All Fields] OR "dysmorphology"[All Fields])
